# Characterization of Equine Rhinitis B Virus Infection in Clinically Ill Horses in the United States during the Period 2012–2023

**DOI:** 10.3390/pathogens12111324

**Published:** 2023-11-07

**Authors:** Chrissie Schneider, Kaitlyn James, Bryant W. Craig, Duane E. Chappell, Wendy Vaala, Philip D. van Harreveld, Cara A. Wright, Samantha Barnum, Nicola Pusterla

**Affiliations:** 1 Merck Animal Health, Rahway, NJ 07065, USAbryant.craig@merck.com (B.W.C.); duane.chappell@merck.com (D.E.C.); vaalanj@aol.com (W.V.); philip.van.harreveld@merck.com (P.D.v.H.); cara.wright@merck.com (C.A.W.); 2 Department of Medicine and Epidemiology, School of Veterinary Medicine, University of California, Davis, CA 95616, USAnpusterla@ucdavis.edu (N.P.)

**Keywords:** equine rhinitis B virus, upper respiratory tract infection, qPCR, equids, viruses, prevalence factors, respiratory tract

## Abstract

Equine rhinitis B virus is a lesser-known equine respiratory pathogen that is being detected with increasing frequency via a voluntary upper respiratory biosurveillance program in the United States. This program received 8684 nasal swab submissions during the years 2012–2023. The nasal swabs were submitted for qPCR testing for six common upper respiratory pathogens: *Streptococcus equi* subspecies *equi* (*S. equi*), equine influenza virus (EIV), equine herpesvirus type 1 (EHV-1), equine herpesvirus type 4 (EHV-4), equine rhinitis A virus (ERAV), and equine rhinitis B virus (ERBV). The overall ERBV qPCR-positivity rate was 5.08% (441/8684). ERBV was detected as a single pathogen in 291 cases (65.99% of positives, 291/441) and was detected as a coinfection with at least one other respiratory pathogen in 150 cases (34.01%, 150/441). Young horses, less than a year of age, with acute onset of fever and respiratory signs and horses used for competition are more likely to test qPCR-positive for ERBV. Horses with ERBV may present with fever, nasal discharge, ocular discharge, and/or cough. Coinfection is a common feature of ERBV infection and *S. equi*, EHV-4 and EIV were the most common pathogens coinfected with ERBV. This report provides important information regarding the clinical relevance of ERBV in the horse and begins investigating the impact of coinfection on clinical disease.

## 1. Introduction

Equine rhinitis B virus (ERBV) is a lesser-known respiratory pathogen in horses which has been detected worldwide over the last 50 years [1,2,3,4,5,6,7,8]. ERBV is a member of the family of Picornaviridae and is the sole virus in the genus Erbovirus. There are currently three known serotypes of ERBV (ERBV1, ERBV2, and ERBV3) [9]. Previous publications report ERBV detection rates of 1.5–30.4% using viral isolation or PCR, and up to 86% using serology [3,4,5,10]. The clinical relevance of ERBV has yet to be fully determined, but it has been detected in horses with clinical signs of acute respiratory disease including fever, nasal discharge, anorexia, cough, lymphadenitis, and limb edema [11]. Previous investigations into ERBV detection reported that ERBV was frequently found along with other viral and bacterial pathogens as coinfections [6,7]. ERBV’s presence as part of a coinfection may contribute to the disease process by lengthening the disease course and/or increasing severity of clinical signs [11]. This report provides information on the demographics, observed clinical signs, and coinfection status of ERBV qPCR-positive horses with clinical respiratory disease in the United States. The objectives of this study are to provide information regarding the clinical relevance of ERBV in horses with respiratory disease and investigate the impact of ERBV coinfection on clinical disease.

## 2. Materials and Methods

### 2.1. Sample Collection, Handling and Processing

Nasal swabs obtained from horses with clinical signs of respiratory disease were submitted by veterinarians enrolled in an ongoing equine respiratory biosurveillance program between September 2012 and April 2023. During this time frame, there were as many as 324 clinics enrolled in the program across 45 states. The submission criteria for this respiratory biosurveillance program included fever (rectal temperature > 101.5 °F, 38.6 °C) and one (or more) of the following clinical signs: nasal discharge, ocular discharge, cough, limb edema, and lethargy. Samples were submitted along with a questionnaire to capture the signalment (age, breed, sex, use), vaccination history, travel history, and clinical signs for each case as reported previously [12]. Submitted samples were tested for *Streptococcus equi* subspecies *equi* (*S. equi*), equine influenza virus (EIV), equine herpesvirus type 1 (EHV-1), equine herpesvirus type 4 (EHV-4), equine rhinitis A virus (ERAV), and ERBV. Additional subsets of this data set have been reviewed in previous publications [12,13,14,15].

Nasal swab samples were collected and shipped overnight on ice to the laboratory. Samples were analyzed using qPCR testing as reported previously [8]. Briefly, on the day of sample arrival to the laboratory, nucleic acid was extracted from the submitted nasal swabs using an automated nucleic acid extraction system (QIAcubeHT, Germantown, MD, USA). cDNA synthesis was performed using the Quantitect Reverse transcription kit (Qiagen, Valencia, CA, USA) following the manufacturer’s directions with slight modifications as previously described [12]. All of the samples were tested for the presence of the housekeeping gene eGAPDH, as previously described, to ensure sample quality and efficiency of nucleic acid extraction [16]. All qPCR testing was performed on submitted samples within 24–48 h of sample collection.

### 2.2. Statistical Analysis

Demographics (including age, breed, sex, and use) and clinical factors (including presence and severity of nasal discharge, ocular discharge, cough, limb edema, anorexia, lethargy, and seasonality) were compared between ERBV qPCR-positive and ERBV qPCR-negative horses using parametric and nonparametric tests, as appropriate. Breed was categorized into Quarter Horse, Thoroughbred, Warmblood, Paint, Arabian, Draft, Pony, and other breed. Use was categorized into competition, pleasure, breeding, other, or unknown. Animal sex was defined as mare, gelding/stallion, or unknown. Age was categorized into less than 1 year of age, 1–4 years, 5–9 years, 10–14 years, 15–19 years, greater than or equal to 20 years, or unknown. Seasons of infection were defined by month groupings. December, January, February were considered winter months; March, April, May were considered spring months; June, July, August were considered summer months; and September, October, November were considered fall months. ERBV qPCR-positive cases were categorized further into ERBV-sole pathogen and ERBV-coinfected; similar comparisons between demographic and clinical factors were conducted between ERBV qPCR-negative, ERBV-sole pathogen, and ERBV-coinfected horses, with *p*-value adjustments for multiple comparisons applied as needed in post hoc tests. Additional descriptive statistics were used to determine the frequency of coinfected pathogens. Statistical significance of infection frequency related to time and season were determined via logistic regression models; odds ratios, 95% confidence intervals, and *p* values were reported where appropriate. For all statistical analyses, a *p* value ≤ 0.05 was considered significant. All analyses were conducted in StataIC, version 16.0.

## 3. Results

### 3.1. ERBV qPCR-Negative vs. ERBV qPCR-Positive

**Figure 1:**
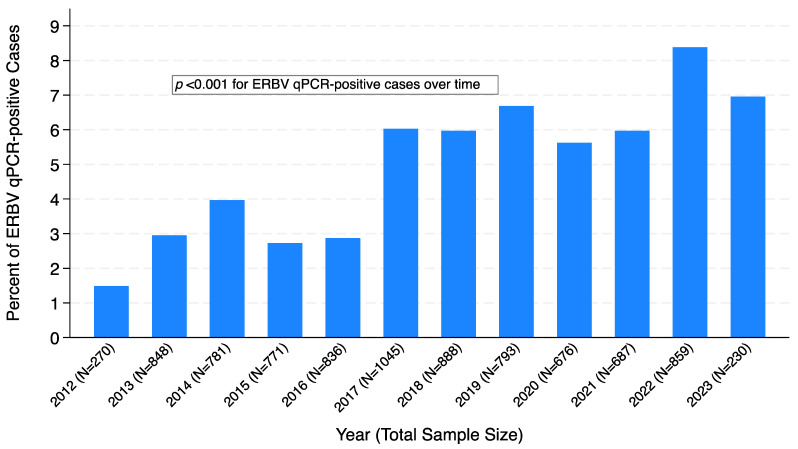
Percent of ERBV qPCR-positive cases per year from 2012–2023. Partial year data available for 2012 and 2023.

**Figure 2:**
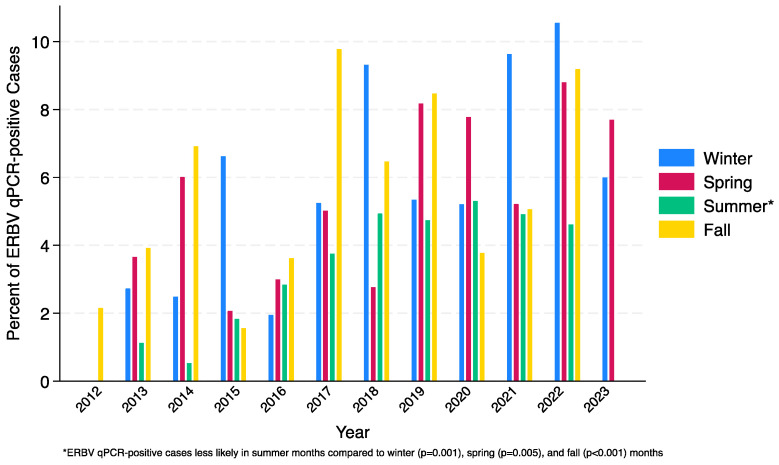
Percent ERBV qPCR-positive cases by season and year.

Nasal swab samples were submitted for 8684 horses between September 2012 and April 2023 from 45 states (Table A1 in Appendix A). A total of 441 samples tested positive for ERBV using qPCR, resulting in an overall positivity rate of 5.08% (441/8684) (Table 1). Throughout the study period there was an increased frequency of ERBV-positive samples (Figure 1). The highest frequency of ERBV in this study was in 2022 at 8.38%, followed by 2023 (partial year) at 6.96%, and 2019 at 6.68%. The frequency of ERBV infections increased over the time period of the biosurveillance cohort collection; the odds ratio for year as a continuous factor was 1.12, 95% CI [1.09, 1.16]; *p* < 0.001. Horses were more likely to be ERBV-positive in the winter, spring, and fall months compared to the summer months (winter versus summer odds ratio 1.65, 95% CI [1.22, 2.25]; *p* = 0.001, spring versus summer odds ratio 1.55, 95% CI [1.14, 2.10]; *p* = 0.005, fall versus summer odds ratio 1.76, 95% CI [1.29, 2.39]; *p* < 0.001) (Figure 2).

There was a significant difference regarding age of horse when comparing ERBV qPCR-positive to ERBV qPCR-negative horses (*p* < 0.001) (Table 1). Horses less than one year of age were significantly more likely to test qPCR-positive for ERBV when compared to horses one year of age and older (*p* < 0.001) (Table 1).

A variety of breeds were represented in the sample submissions, including Quarter Horse (QH) representing 37.2% of sample submissions, Thoroughbred (TB) at 15.5%, Warmblood (WB) at 10%, Paint at 4%, Arabian at 6%, Draft at 2.8%, Pony at 3.9%, and other breed comprising 20.7% of sample submissions. The global *p* value indicated a significant statistical difference regarding breed of horse when comparing ERBV qPCR-positive to ERBV qPCR-negative (*p* < 0.001), but upon post hoc testing with multiple comparison adjustment, only the Pony breed had a different distribution between ERBV qPCR-positive versus ERBV qPCR-negative using our Bonferroni adjusted *p*-value threshold (Table 1).

There was not a significant difference in ERBV positivity status between the different sexes (mare, gelding/stallion, unknown).

There was a significant difference regarding the use of the horse when comparing ERBV qPCR-positive to ERBV qPCR-negative (*p* < 0.001) (Table 1). Horses used for competition purposes were more likely to be ERBV qPCR-positive (*p* < 0.001) and horses used for pleasure riding were more likely to be ERBV qPCR-negative (*p* < 0.001) (Table 1). The majority of ponies in this dataset were used for pleasure (*n* = 218/302, 72%, data not shown). The other use categories included in the sample set were breeding, other, and unknown (i.e., use not indicated on the sample submission form).

**Figure 3:**
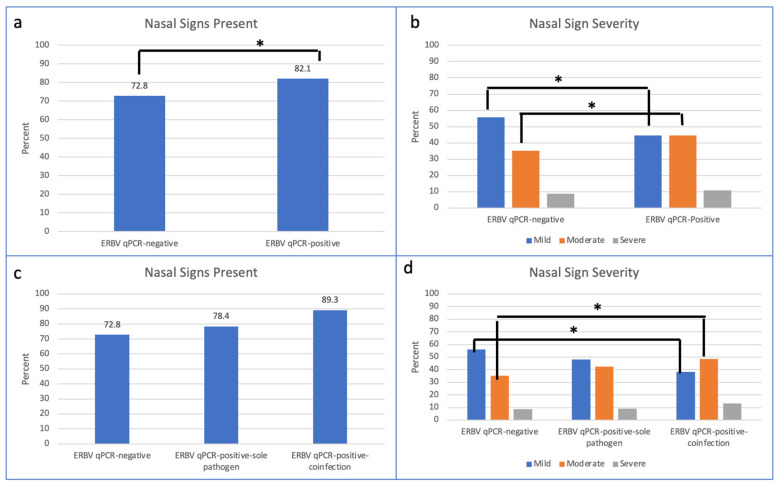
Presence and severity of nasal discharge in (**a**,**b**) ERBV qPCR-negative and ERBV qPCR-positive and (**c**,**d**) ERBV qPCR-negative, ERBV qPCR-positive–sole pathogen, and ERBV qPCR-positive–coinfection. * *p* < 0.05.

Nasal discharge (*p* < 0.001), ocular discharge (*p* = 0.014), and cough (*p* = 0.006) were the clinical signs associated with ERBV detection (Table 2). Of the horses presenting with nasal discharge, those with moderate nasal discharge were more likely to be ERBV qPCR-positive (*p* < 0.001) (Table 2) (Figure 3). Of the horses with ocular discharge, those with moderate ocular discharge were more likely to be ERBV qPCR-positive (*p* = 0.004) (Table 2).

### 3.2. ERBV qPCR-Negative vs. ERBV qPCR-Positive Sole Pathogen vs. ERBV qPCR-Positive Coinfection

Of the ERBV qPCR-positive samples, ERBV was the sole pathogen detected in 291 samples (65.99%, 291/441) and was detected as part of a coinfection with at least one other respiratory pathogen in 150 samples (36.28%, 150/441) (Table 3). The majority of ERBV coinfections were with *S*. *equi* (58.0% of coinfections), EHV-4 (32.0%), and EIV (16.7%) (Table 4).

Horses less than a year of age were more likely to be ERBV qPCR-positive, but there was not a difference in that age group between ERBV qPCR-positive as a sole pathogen and ERBV qPCR-positive as a coinfection (Table 3). Age was not a significant factor when comparing ERBV-sole pathogen vs. ERBV-coinfection (Table 3).

The global *p* value showed a significant difference regarding breed of horse when comparing ERBV qPCR-negative, ERBV qPCR-positive–sole pathogen, and ERBV qPCR-positive–coinfection (*p* = 0.028), but there was not a significant difference in post hoc testing (Table 3).

No significant difference was observed between ERBV qPCR-negative, ERBV qPCR-positive–sole pathogen, and ERBV qPCR-positive–coinfection with respect to sex (female, gelding/stallion, or unknown) (Table 3).

A horse’s use did not affect whether its ERBV infection was a sole pathogen or part of a coinfection (Table 3).

Horses with ERBV qPCR-positive–coinfection were significantly more likely to present with nasal discharge and cough when compared to ERBV qPCR-negative cases (*p* < 0.001) and ERBV qPCR-positive–sole pathogen cases (*p* = 0.01) (Table 5). Of the horses that presented with nasal discharge, the horses with ERBV qPCR-positive–coinfection were more likely to have moderate or severe nasal discharge when compared to ERBV qPCR-negative cases (Table 5). There was no difference in severity of nasal discharge detected between ERBV qPCR-positive–sole pathogen cases and ERBV qPCR-positive–coinfection cases (Figure 3).

There was not a significant difference between ERBV qPCR-negative, ERBV qPCR-positive–sole pathogen, and ERBV qPCR-positive–coinfection horses with respect to the presence of fever, ocular discharge, limb edema, anorexia, and lethargy (Table 5).

## 4. Discussion

The frequency of ERBV in this study’s population during the 10-year-and-8-month time period evaluated was 5.08%. The increasing frequency of ERBV detection over the study period indicates that clinicians are more likely to be presented with an ERBV clinically infected case than in the past. This fact makes it even more important to characterize ERBV infection in horses and help practitioners interpret ERBV diagnostics.

ERBV infection demonstrated a consistent seasonality difference across all the years included in the study cohort. Infections were less common in summer months compared to other times of year. The reason for this is speculative and may relate to the age, husbandry, and use of sampled horses [14]. While there were ERBV cases in every age category in this study, horses less than one year of age were over-represented. This may be due to young horses’ immature immune system. Perhaps these young horses show more obvious clinical signs the first time their immune system is exposed to ERBV leading to an examination and diagnostic testing. Horses used for competition were also more likely to be ERBV qPCR-positive, which may be due to increased exposure to ERBV, along with other respiratory pathogens, when co-mingled with horses from other farms at events. While the Pony breed did demonstrate a lower frequency of ERBV positive samples, we believe this is not necessarily due to genetics but potentially a factor of use distribution and management strategies. A horse’s sex did not play a role in their susceptibility to ERBV infection.

Historically, the clinical significance of an ERBV qPCR-positive result has remained poorly characterized. Therefore, clinicians presented with a horse displaying acute signs of respiratory disease may choose to ignore an ERBV qPCR-positive result, concluding that the detection of ERBV in their case isn’t clinically relevant. The findings of the present study do not support that conclusion as multiple clinical signs were associated with an ERBV qPCR-positive nasal swab. Horses with ERBV infection (as a sole pathogen or as part of a coinfection) displayed fever, nasal discharge, ocular discharge, and cough. Ocular discharge may be a clinical sign of respiratory disease that is sometimes overlooked by practitioners and this study highlights the relevance of this clinical entity.

In this study, it was common for ERBV to be part of a coinfection with at least one other respiratory pathogen. This phenomenon has been reported previously and it has been hypothesized that, as part of a coinfection, ERBV may contribute to worsened severity of disease [11]. This theory was not supported by the results of the present study. There was not a significant difference in the severity of any of the reported clinical sign between ERBV qPCR-positive–sole pathogen cases and ERBV qPCR-positive–coinfection cases. One variable that could not be controlled in this study is the role of vaccination against the pathogens commonly coinfected with ERBV, specifically *S. equi*, EHV-4, and EIV. Whether a horse had been vaccinated against these pathogens, and when, could have affected the severity of clinical signs contributed by those pathogens. Another variable that could affect the severity of clinical signs reported is timing of sampling during the course of disease. In this study, samples were submitted at one single time point for each case, which could affect both the likelihood of detecting a pathogen and also the presence/severity of clinical signs at the time of sampling (early in disease vs. later in disease).

Another theory that has been suggested is that ERBV coinfection may increase the length of time a horse displays the clinical signs of respiratory disease [11]. This theory could not be evaluated in this study as cases were not followed over time. Further, acute and convalescent serum samples were not available for ERBV serology in order to support recent infection.

Limitations of this study include that the samples were voluntary sample submissions from horses with respiratory disease. No control or normal horses were sampled as part of this study and cases were not followed over time. In addition, the nasal swab sampling method may have impacted our results as other sampling methods may increase the likelihood of detecting various pathogens.

There is much left to learn regarding ERBV and its role in equine health. Future studies could investigate the length of disease course, persistence of ERBV infection, tissues infected by ERBV, reservoirs of ERBV in the equine population, and methods of prevention. There is more to learn regarding the three known serotypes of ERBV as well. This study investigated the presence of any ERBV RNA in nasal secretions via qPCR and serotypes were not determined. The virulence, prevalence, and clinical role of the ERBV serotypes have yet to be fully detailed.

In conclusion, ERBV frequency is increasing overall, and ERBV plays a clinically relevant role in equine respiratory disease. Young horses with acute onset of fever and respiratory signs, that are less than a year of age, and horses used for competition are more likely to test qPCR-positive for ERBV. Horses with ERBV may present with fever, nasal discharge, ocular discharge and/or cough. Coinfection is a common feature of ERBV infection and *S*. *equi*, EHV-4 and EIV were the most common pathogens coinfected with ERBV.

## Figures and Tables

**Table 1:** Demographic information for horses testing ERBV qPCR-negative and ERBV qPCR-positive.

	Total Cohort (N = 8684)	ERBV Negative (*n* = 8243)	ERBV Positive (*n* = 441)	*p*-Value *
***Age (in years)***				*<0.001*
<1	1369 (15.8%)	1203 (14.6%)	166 (37.6%)	<0.0001
1–4	2161 (24.9%)	2043 (24.8%)	118 (26.8%)	NS
5–9	1809 (20.8%)	1754 (21.3%)	55 (12.5%)	<0.0001
10–14	1320 (15.2%)	1287 (15.6%)	33 (7.5%)	<0.0001
15–19	814 (9.4%)	797 (9.7%)	17 (3.9%)	<0.0001
20+	591 (6.8%)	569 (6.9%)	22 (5.0%)	NS
Unknown	620 (7.1%)	590 (7.2%)	30 (6.8%)	--
***Breed***				*<0.001*
Quarter horse	3228 (37.2%)	3049 (37.0%)	179 (40.6%)	NS
Thoroughbred	1344 (15.5%)	1259 (15.3%)	85 (19.3%)	NS
Warmblood	866 (10.0%)	830 (10.1%)	36 (8.2%)	NS
Paint	351 (4.0%)	338 (4.1%)	13 (2.9%)	NS
Arabian	518 (6.0%)	480 (5.8%)	38 (8.6%)	NS
Draft	241 (2.8%)	230 (2.8%)	11 (2.5%)	NS
Pony	339 (3.9%)	331 (4.0%)	8 (1.8%)	0.001
Other	1797 (20.7%)	1726 (20.9%)	71 (16.1%)	NS
***Sex***				*0.64*
Mare	3078 (35.4%)	2919 (35.4%)	159 (36.1%)	
Gelding/Stallion	4487 (51.7%)	4266 (51.8%)	221 (50.1%)	
Unknown	1119 (12.9%)	1058 (12.8%)	61 (13.8%)	
***Use***				*<0.001*
Competition	3436 (39.6%)	3222 (39.1%)	214 (48.5%)	0.0001
Pleasure	3217 (37.0%)	3101 (37.6%)	116 (26.3%)	<0.0001
Breeding	407 (4.7%)	385 (4.7%)	22 (5.0%)	NS
Other	549 (6.3%)	526 (6.4%)	23 (5.2%)	NS
Unknown	1075 (12.4%)	1009 (12.2%)	66 (15.0%)	--

* Factors with global *p*-values (italicized) that met the statistical significance threshold of <0.05 underwent post hoc testing to determine differences, excluding the unknown categories. For age and breed, the Bonferroni corrected *p*-value threshold of significance was *p* < 0.008. For use, the Bonferroni corrected *p*-value threshold of significance was *p* < 0.0125. NS = not significant.

**Table 2:** Reported frequency and severity of clinical signs for horses testing ERBV qPCR-negative and ERBV qPCR-positive.

	Total Cohort (N = 8684)	ERBV Negative (*n* = 8243)	ERBV Positive (*n* = 441)	*p*-Value *
**Presence, any severity**				
Nasal discharge	6366 (73.3%)	6004 (72.8%)	362 (82.1%)	<0.001
Ocular signs	2115 (24.4%)	1982 (24.0%)	133 (30.2%)	0.014
Cough	4149 (47.8%)	3909 (47.4%)	240 (54.4%)	0.006
Limb edema	870 (10.0%)	834 (10.1%)	36 (8.2%)	0.22
Anorexia	5232 (60.2%)	4956 (60.1%)	276 (62.6%)	0.57
Lethargy	6331 (72.9%)	6006 (72.9%)	325 (73.7%)	0.40
Fever (>101.5)	7376 (84.9%)	6988 (84.8%)	388 (88.0%)	0.44
**Severity of signs**				
Nasal discharge				*<0.001*
Mild	3515 (55.2%)	3354 (55.9%)	161 (44.5%)	<0.0001
Moderate	2281 (35.8%)	2119 (35.3%)	162 (44.7%)	0.0003
Severe	570 (8.9%)	531 (8.8%)	39 (10.8%)	NS
Ocular symptoms				*0.014*
Mild	1688 (79.8%)	1593 (80.4%)	95 (71.4%)	0.0123
Moderate	392 (18.5%)	355 (17.9%)	37 (27.8%)	0.0044
Severe	35 (1.65%)	34 (1.7%)	1 (0.75%)	NS

* Factors with global *p*-values (italicized) that met the statistical significance threshold of <0.05 underwent post hoc testing to determine differences, excluding the unknown categories. For severity of clinical signs, the Bonferroni corrected *p*-value threshold of significance was *p* < 0.017. NS = not significant.

**Table 3:** Demographic information for horses testing ERBV qPCR-negative, ERBV qPCR-positive–sole pathogen, and ERBV qPCR-positive coinfection.

	ERBV-Negative (*n* = 8243)	ERBV-Sole Pathogen (*n* = 291)	ERBV-Coinfection (*n* = 150)	*p*-Value: ERBV-Negative vs. ERBV-Sole Pathogen *	*p*-Value: ERBV-Negative vs. ERBV-Coinfection *	*p*-Value: ERBV-Sole Pathogen vs. ERBV-Coinfection *
**Age (in years)**				*<0.001*	*<0.001*	*0.26*
<1	1203 (14.6%)	111 (38.1%)	55 (36.7%)	<0.0001	<0.0001	
1–4	2043 (24.8%)	80 (27.5%)	38 (25.3%)	NS	NS	
5–9	1754 (21.3%)	30 (10.3%)	25 (16.7%)	<0.0001	NS	
10–14	1287 (15.6%)	26 (8.9%)	7 (4.7%)	0.002	0.0002	
15–19	797 (9.7%)	12 (4.1%)	5 (3.3%)	0.001	0.0079	
20+	569 (6.9%)	13 (4.5%)	9 (6.0%)	NS	NS	
Unknown	590 (7.2%)	19 (6.5%)	11 (7.3%)	--	NS	
**Breed**				*0.028*	*0.065*	*0.75*
Quarter horse	3049 (37.0%)	114 (39.2%)	65 (43.3%)	NS		
Thoroughbred	1259 (15.3%)	60 (20.6%)	25 (16.7%)	NS		
Warmblood	830 (10.1%)	26 (8.9%)	10 (6.7%)	NS		
Paint	338 (4.1%)	9 (3.1%)	4 (2.7%)	NS		
Arabian	480 (5.8%)	22 (7.6%)	16 (10.7%)	NS		
Draft	230 (2.8%)	8 (2.7%)	3 (2.0%)	NS		
Pony	331 (4.0%)	4 (1.4%)	4 (2.7%)	NS		
Other	1726 (20.9%)	48 (16.5%)	23 (15.3%)	--		
**Sex**				*0.96*	*0.37*	*0.45*
Mare	2919 (35.4%)	102 (35.1%)	57 (38.0%)	NS		
Gelding/Stallion	4266 (51.8%)	150 (51.5%)	71 (47.3%)	NS		
Unknown	1058 (12.8%)	39 (13.4%)	22 (14.7%)	--		
**Use**				*<0.001*	*0.053*	*0.76*
Competition	3222 (39.1%)	142 (48.8%)	72 (48.0%)	0.0009		
Pleasure	3101 (37.6%)	73 (25.1%)	43 (28.7%)	<0.0001		
Breeding	385 (4.7%)	14 (4.8%)	8 (5.3%)	NS		
Other	526 (6.4%)	17 (5.8%)	6 (4.0%)	NS		
Unknown	1009 (12.2%)	45 (15.5%)	21 (14.0%)	--		

* Factors with global *p*-values (italicized) that met the statistical significance threshold of <0.05 underwent post hoc testing to determine differences, excluding the unknown categories. For age and breed, the Bonferroni corrected *p*-value threshold of significance was *p* < 0.008. For use, the Bonferroni corrected *p*-value threshold of significance was *p* < 0.0125. NS = not significant.

**Table 4:** Frequency of pathogens detected as part of an ERBV qPCR-positive–coinfection compared to ERBV qPCR-negative cases.

	Total Cohort (N = 8684) *	ERBV-Negative (*n* = 8243)	ERBV-Coinfection (*n* = 150)	*p*-Value
**qPCR positive co-infections**				
EHV-1 (blood)	40 (0.5%)	39 (0.5%)	1 (0.7%)	0.56
EHV-1 (nasal)	114 (1.3%)	110 (1.3%)	4 (1.0%)	0.05
EHV-4	939 (10.8%)	891 (10.8%)	48 (32.0%)	<0.001
*S. equi* subspecies *equi*	855 (9.9%)	768 (9.3%)	87 (58.0%)	<0.001
ERAV	14 (0.2%)	13 (0.2%)	1 (0.7%)	0.24
EIV	936 (10.8%)	911 (11.1%)	25 (16.7%)	<0.001

* Total cohort includes ERBV qPCR positive-sole pathogen.

**Table 5:** Reported clinical signs for horses testing ERBV qPCR-negative, ERBV qPCR-positive–sole pathogen, and ERBV qPCR-positive–coinfection.

	ERBV-Negative (*n* = 8243)	ERBV-Sole Pathogen (*n* = 291)	ERBV-Coinfection (*n* = 150)	*p*-Value: ERBV-Negative vs. ERBV-Sole pathogen *	*p*-Value: ERBV-Negative vs. ERBV-Coinfection *	*p*-Value: ERBV-Sole Pathogen vs. ERBV-Coinfection *
**Presence, any severity**						
Nasal discharge	6004 (72.8%)	228 (78.4%)	134 (89.3%)	0.11	<0.001	0.01
Ocular signs	1982 (24.0%)	86 (29.6%)	47 (31.3%)	0.09	0.12	0.93
Cough	3909 (47.4%)	145 (49.8%)	95 (63.3%)	0.41	<0.001	0.014
Limb edema	834 (10.1%)	24 (8.2%)	12 (8.0%)	0.56	0.20	0.43
Anorexia	4956 (60.1%)	179 (61.5%)	97 (64.7%)	0.83	0.53	0.77
Lethargy	6006 (72.9%)	211 (72.5%)	114 (76.0%)	0.43	0.63	0.68
Fever (>101.5)	6988 (84.8%)	46 (86.8%)	342 (88.1%)	0.80	0.09	0.18
**Severity of signs**						
Nasal discharge				*0.06*	*<0.001*	*0.133*
Mild	3355 (55.9%)	110 (48.2%)	51 (38.1%)		<0.0001	
Moderate	2119 (35.3%)	97 (42.5%)	65 (48.5%)		0.0008	
Severe	531 (8.84%)	21 (9.2%)	18 (13.4%)		NS	
Ocular signs				*0.06*	*0.11*	*0.36*
Mild	1594 (80.4%)	63 (73.3%)	32 (68.1%)			
Moderate	355 (17.9%)	23 (26.7%)	14 (29.8%)			
Severe	34 (1.7%)	0 (0.0%)	1 (2.1%)			

* Factors with global *p*-values (italicized) that met the statistical significance threshold of <0.05 underwent post hoc testing to determine differences, excluding the unknown categories. For severity of clinical signs, the Bonferroni corrected *p*-value threshold of significance was *p* < 0.017. NS = not significant.

## Data Availability

Data are available upon request due to privacy restrictions.

## Appendix A

**Table A1:** Frequency of case submissions from the United States, 2012–2023.

State	Number of Submissions (N, % of Total)
AL	28 (0.54%)
AR	6 (0.12%)
AZ	164 (3.17%)
CA	189 (3.65%)
CO	166 (3.21%)
CT	1 (0.02%)
FL	225 (4.35%)
GA	61 (1.18%)
IA	51 (0.99%)
ID	11 (0.21%)
IL	3 (0.06%)
IN	6 (0.12%)
KS	20 (0.39%)
KY	92 (1.78%)
KZ	3 (0.06%)
LA	2 (0.04%)
MD	81 (1.57%)
MI	77 (1.49%)
MN	85 (1.64%)
MO	188 (3.63%)
MP	2 (0.04%)
MS	1 (0.02%)
MT	22 (0.43%)
NC	67 (1.3%)
NE	87 (1.68%)
NJ	8 (0.15%)
NV	5 (0.1%)
NY	262 (5.06%)
OH	251 (4.85%)
OK	43 (0.83%)
OR	198 (3.83%)
PA	315 (6.09%)
RI	124 (2.4%)
SC	237 (4.58%)
SD	11 (0.21%)
TN	553 (10.69%)
TX	1045 (20.2%)
UT	1 (0.02%)
VA	133 (2.57%)
VT	1 (0.02%)
WA	213 (4.12%)
WI	100 (1.93%)
WV	6 (0.12%)
WY	29 (0.56%)
